# Hepatocellular carcinoma in a large medical center of China over a 10-year period: evolving therapeutic option and improving survival

**DOI:** 10.18632/oncotarget.2913

**Published:** 2015-02-24

**Authors:** Qianqian Zhu, Na Li, Xiaoyan Zeng, Qunying Han, Fang Li, Cuiling Yang, Yi Lv, Zhihua Zhou, Zhengwen Liu

**Affiliations:** ^1^ Department of Infectious Diseases, First Affiliated Hospital, School of Medicine, Xi’an Jiaotong University, Xi’an, 710061 Shaanxi, China; ^2^ Department of Laboratory Medicine, First Affiliated Hospital, School of Medicine, Xi’an Jiaotong University, Xi’an, 710061 Shaanxi, China; ^3^ Department of Hepatobiliary Surgery, First Affiliated Hospital, School of Medicine, Xi’an Jiaotong University, Xi’an, 710061 Shaanxi, China; ^4^ Institute of Advanced Surgical Technology and Engineering, Xi’an Jiaotong University, Xi’an, 710061 Shaanxi, China

**Keywords:** hepatocellular carcinoma, feature, treatment, survival, diagnosis

## Abstract

**Background:**

Hepatocellular carcinoma (HCC) is among the most common and lethal cancers worldwide, especially in China.

**Methods:**

We retrospectively analyzed data from patients who were diagnosed and treated HCC between 2002 and 2011 in a large hospital in northwest China and compared the data between periods 2002–2006 (P1) and 2007–2011 (P2).

**Results:**

2045 patients were included in analysis. The HCC stages at diagnosis according to the Barcelona clinic liver cancer staging system had no significant change. Treatment options of liver transplantation, transcatheter arterial chemoembolization and other therapy decreased while percutaneous local ablation and supportive care increased from P1 to P2. Options of surgical resection and systematic therapy had no significant change. Patient survival rates at 1, 3 and 5 years significantly improved from P1 to P2. The treatments with increasing option trend had a higher magnitude of survival increase and *vise versa*.

**Conclusion:**

Over the last 10 years, the patient survival had a significant increase which was mainly a result of the optimal therapeutic selections according to disease stages in this center. However, the proportion of patients diagnosed at early stages of HCC remained low and did not increase, a result calling for implementing surveillance system for at risk patients.

## INTRODUCTION

Hepatocellular carcinoma (HCC) is one of the most common cancers leading to cancer-related death [1]. Liver cirrhosis is a risk factor for HCC. Hepatitis B virus (HBV) and hepatitis C virus (HCV) infections are major causes of liver cirrhosis and HCC [2, 3] though the impact of HBV is declining due to hepatitis B vaccination [4].

The prognoses of patients with HCC are determined by liver function, general health status, tumor status, and efficacy of treatment [5]. Management of HCC is based on the tumor location and size, liver function, and the performance status of patients [6]. The important effecting factors for long-term survival in HCC patients are to diagnose the tumor at an early stage, and treat the patients with effective therapies [7, 8]. In order to diagnose the patients at an early stage, surveillance is a key effector for the patients at high HCC risk [9]. Patients diagnosed at an early stage have a high chance of curative treatment and improved long-term survival [8]. Beyond surveillance, an effective prognostic system is very important for its guide role for therapy options [6, 10]. The uni-dimensional prognostic systems, such as Child-Pugh score, and the tumor node metastases classification, may result in inaccurate survival prediction of HCC because of their lacking the assessment of prognostic effectors [10]. Based on tumor status, liver functional status and the performance status of patients, the Barcelona clinic liver cancer (BCLC) staging classification has been proved a good prognostic system and has been recommended by the American Association for the Study of Liver Disease (ASSLD) [10–12]. Multiple therapeutics such as surgical resection, transcatheter arterial chemoembolization (TACE), percutaneous local ablation and systematic therapy are available for HCC and the efficacy may vary with the options of treatments and the cancer staging. Moreover, the therapeutic strategy and the option rationale may evolve with the advent of novel treatment, the improvement of therapeutic expertise and the deepening to the understanding of HCC features.

China has a high incidence of HCC. However, to the best of our knowledge, it remains largely unclear whether there have been changes in the characteristics, diagnosis, treatment and prognosis of HCC over time and whether there are identifiable contributing factors to the possible improvement in the management of HCC. To this end, this study was carried out based on the analysis of data from a large university hospital in northwest China over a ten-year period.

## RESULTS

### Overall characteristics of patients

A total of 2745 patients diagnosed with HCC were reviewed. Because of the possible affect on HCC associated survival analysis, 570 patients who were not firstly diagnosed and treated as HCC in our hospital, 76 patients with other cancers at the same time and 54 patients with other serious diseases were excluded. The remaining 2045 patients were included in further analysis. The average follow-up time was 43.6 months. Of the 2045 patients, 558 (27.3%) and 1487 (72.7%) patients were diagnosed and treated during periods 2002–2006 (P1) and 2007–2011 (P2), respectively (Table 1). A total of 1494 patients died of HCC at the end of follow-up.

**Table 1:** Demographics, etiology and biochemistry of liver disease and Child-Pugh class of patients

	Total	2002–2006	2007–2011	*p*
n (%)	2045 (100)	558 (27.3)	1487 (72.7)	
Gender [male/female (%)]	1662/383 (81.3/18.7)	453/105 (81.2/18.8)	1209/278 (81.3/18.7)	0.950
Age (mean ± SD)	2045 (52.5 ± 12.1)	51.5 ± 12.5	53.0 ± 12.0	0.017
Etiology [n (%)]	2045 (100)			0.003
HBV	1504 (73.5)	396 (71.0)	1108 (74.5)	0.106
HCV	83 (4.1)	15 (2.7)	68 (4.6)	0.054
HBV+HCV	17 (0.8)	2 (0.4)	15 (1.0)	0.242
The others	441 (21.6)	145 (26.0)	296 (19.9)	0.003
ALT [n (%)]	2045 (100)			0.076
≤ 40	783 (38.3)	225 (40.3)	558 (37.5)	
40–200	1171 (57.3)	301 (53.9)	870 (58.5)	
>200	91 (4.4)	32 (5.7)	59 (4.0)	
AST [n (%)]	2045 (100)			0.633
≤ 40	401 (19.6)	104 (18.6)	297 (20.0)	
40–200	1399 (68.4)	382 (68.5)	1017 (68.4)	
>200	245 (12.0)	72 (12.93)	173 (11.6)	
Child-Pugh class [n (%)]	1843 (90.1)			0.587
A	959 (52.0)	266 (53.8)	693 (51.4)	
B	771 (41.8)	197 (39.9)	574 (42.6)	
C	113 (6.1)	31 (6.3)	82 (6.1)	

SD, standard deviation; HBV, hepatitis B virus; HCV, hepatitis C virus; ALT, alanine aminotransferase; AST, aspartate aminotransferase.

Of the 2045 patients, 1662 (81.3%) were male with no significant gender ratio difference between P1 and P2. The mean age of patients diagnosed as HCC increased from 51.5 ± 12.5 years in P1 to 53.0 ± 12.0 years in P2 (*p* = 0.017, Table 1).

There were 1504 (73.5%) HBV infections, 83 (4.1%) HCV infections and 17 (0.8%) co-infected with HBV and HCV (Table 1). The etiology of HCC had different distribution between P1 and P2 with HCV infection being increased (*p* = 0.054), HBV and HBV+HCV no significant change and other causes significantly decreased (*p* = 0.003). HBV infection was the main risk factor for each period (Table 1).

The liver transaminase profile and Child-Pugh class in the patients had no significant difference between P1 and P2 (Table 1).

### HCC features

The α-fetoprotein (AFP) levels were markedly elevated in 57.4% of the patients and had no significant difference between patients in P1 and P2 (Table 2).

**Table 2:** Features of hepatocellular carcinoma

	***n* (%)**	**2002–2006**	**2007–2011**	***p***
a-fetoprotein	1489 (72.8)			0.492
≤ 20	374 (25.1)	119 (26.2)	255 (24.7)	
20–200	261 (17.5)	72 (15.8)	189 (18.3)	
>200	854 (57.4)	264 (58.0)	590 (57.1)	
BCLC stage	1773 (86.7)			0.316
Very early	32 (1.8)	6 (1.3)	26 (2.0)	
Early	303 (17.1)	69 (14.6)	234 (18.0)	
Intermediate	1128 (63.6)	308 (65.1)	820 (63.1)	
Advanced	197 (11.1)	59 (12.5)	138 (10.6)	
Terminal	113 (6.4)	31 (6.6)	82 (6.3)	
Metastasis	2045 (100)			0.147
Intrahepatic	118 (5.8)	23 (4,1)	95 (6.4)	
Extrahepatic	216 (10.6)	60 (10.8)	156 (10.5)	
No	1711 (83.7)	475 (85.1)	1236 (83.1)	

There were 272 (13.3%) patients who could not be classified by BCLC staging system because of non-cirrhotic or insufficient data. Among the patients who could be classified, 32 (1.8%), 303 (17.1%), 1128 (63.6%), 197 (11.1%) and 113 (6.4%) patients were classified as BCLC very early, early, intermediated, advanced, and terminal stages, respectively, with no difference between P1 and P2 (Table 2).

Patients of 16.4% had metastasis at diagnosis with no difference between P1 and P2 (Table 2).

### Treatment options

**Figure 1:**
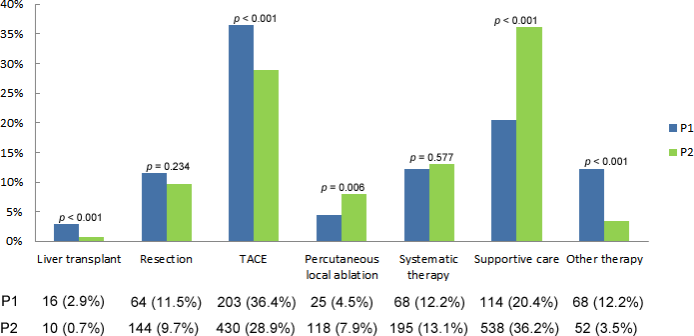
Treatments of hepatocellular carcinoma in the two considered periods 2002–2006 (P1) and 2007–2011 (P2) TACE, transcatheter arterial chemoembolization.

Supportive care and TACE were the most common treatment options in the whole period of study (31.9% and 31.0%, respectively). The treatment options had some changes from P1 to P2. Liver transplantation decreased from 2.9% in P1 to 0.7% in P2 (*p* < 0.001). Other main differences of treatment options were the reduction in TACE (36.4% vs. 28.9%, *p* < 0.001) and other therapy (12.2% vs. 3.5%, *p* < 0.001) and the increase in percutaneous local ablation (4.5% vs. 7.9%, *p* = 0.006) and supportive care (20.4% vs. 36.2%, *p* < 0.001) between P1 and P2. Surgical resection and systematic therapy had no significant change between the 2 periods (Figure 1).

### Survival rates

**Figure 2:**
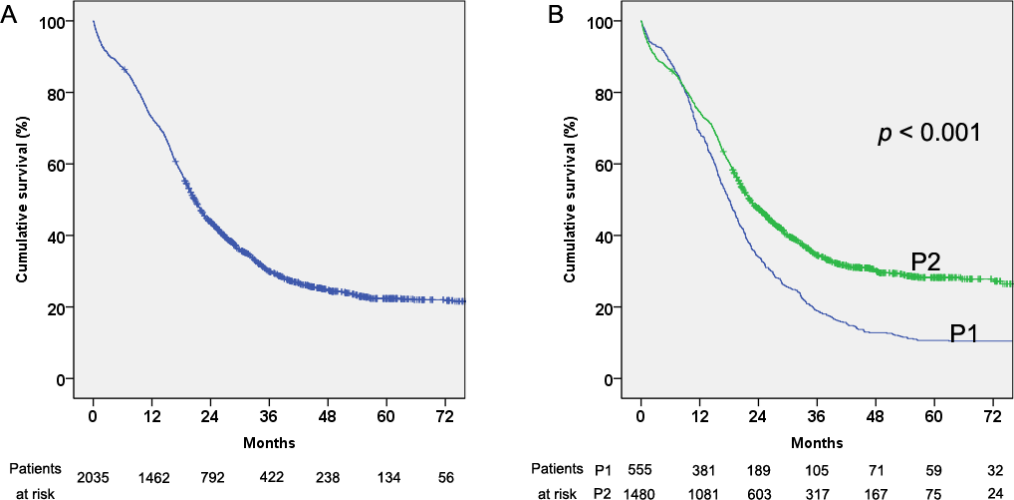
Cumulative survival of all the hepatocellular carcinoma (HCC) patients and in the two considered periods 2002–2006 (P1) and 2007–2011 (P2) **(A)** Cumulative survival of all the HCC patients. **(B)** Cumulative survival of all the HCC patients in the two considered periods 2002–2006 (P1) and 2007–2011 (P2).

The overall 1-, 3- and 5-year survival rates of the 2045 patients were 44%, 25% and 22%, respectively (Figure 2A). The survival rates at 1, 3 and 5 years increased significantly from P1 (34%, 13% and 10%, respectively) to P2 (47%, 30% and 28%, respectively, *p* < 0.001, Figure 2B).

**Figure 3:**
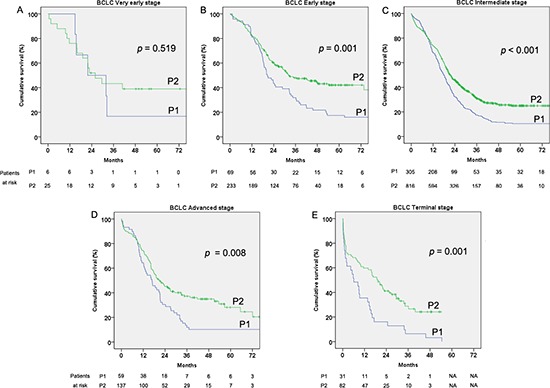
Cumulative survival of the hepatocellular carcinoma (HCC) patients by Barcelona clinic liver cancer (BCLC) staging classification in the two considered periods 2002–2006 (P1) and 2007–2011 (P2) **(A)** Very early stage. **(B)** Early stage. **(C)** Intermediate stage. **(D)** Advanced stage. **(E)** Terminal stage.

A significant survival increasing trend can be seen in almost each BCLC, except very early, stage from P1 to P2 (*p* = 0.519, *p* = 0.001, *p* < 0.001, *p* = 0.008 and *p* = 0.001 for BCLC very early stage, early stage, intermediate stage, advanced stage and terminal stage, respectively, Figure 3A–3E).

**Figure 4:**
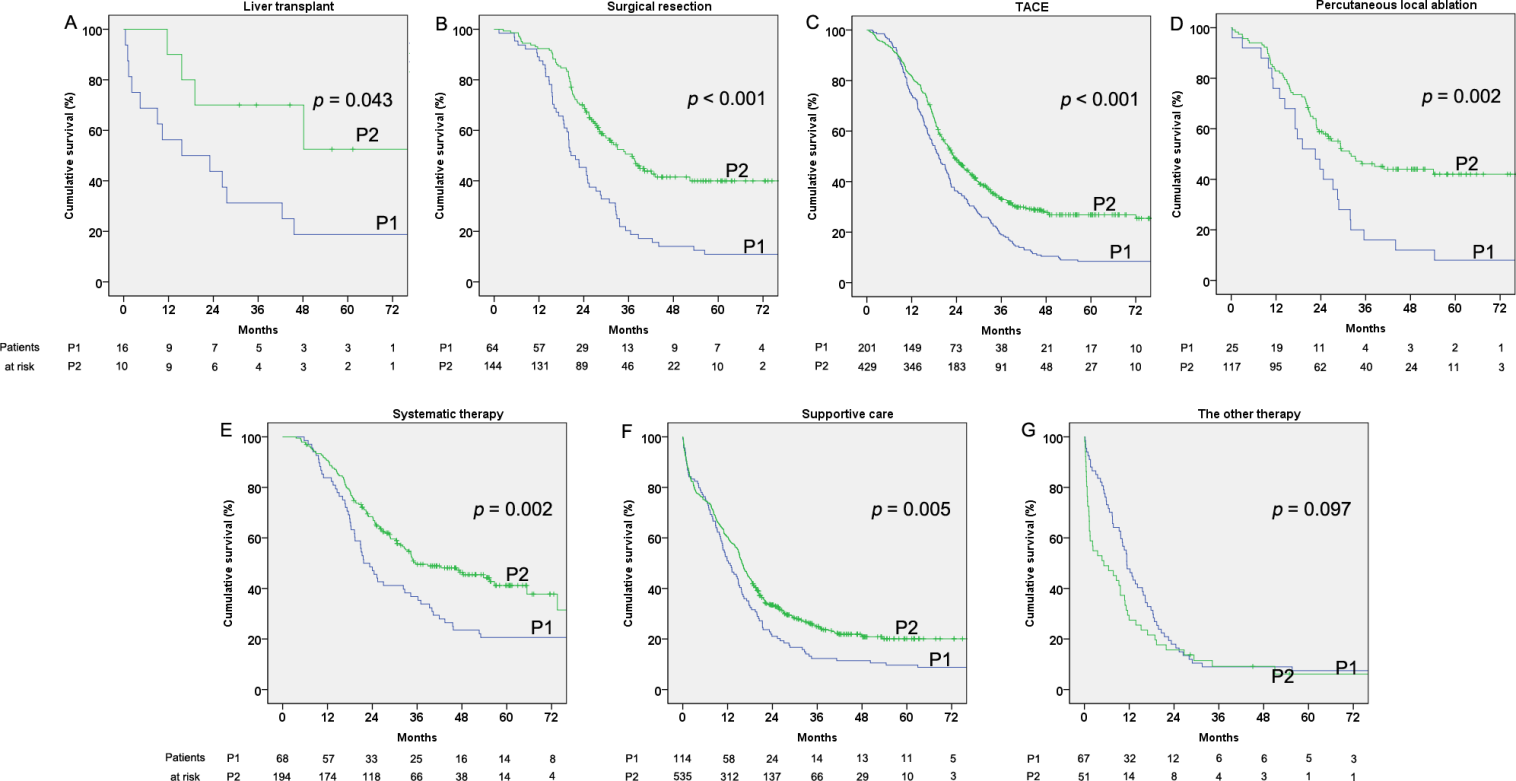
Cumulative survival of the hepatocellular carcinoma (HCC) patients by treatment in the two considered periods 2002–2006 (P1) and 2007–2011 (P2) **(A)** Liver transplantation. **(B)** Surgical resection. **(C)** Transcatheter arterial chemoembolization (TACE). **(D)** Percutaneous local ablation. **(E)** Systematic therapy. **(F)** Supportive care. **(G)** Other therapy.

A significant survival increasing trend can also be seen in almost each treatment, except other therapy, from P1 to P2 (*p* = 0.043, *p* < 0.001, *p* < 0.001, *p* = 0.002, *p* = 0.002, *p* = 0.005 and *p* = 0.097 for liver transplantation, resection, TACE, percutaneous local ablation, systematic therapy, supportive care and the other therapy, respectively, Figure 4A–4G).

**Figure 5:**
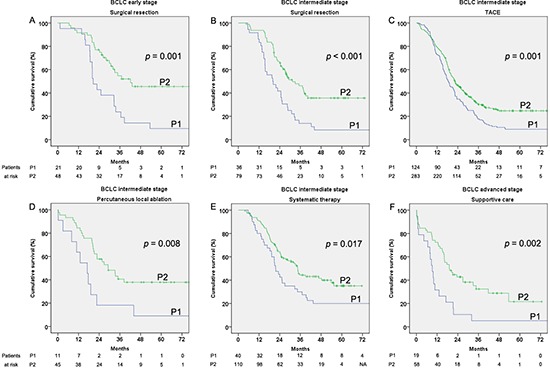
Cumulative survival of patients by treatments which showed increased survival according Barcelona clinic liver cancer (BCLC) staging classification stages in the two considered periods 2002–2006 (P1) and 2007–2011 (P2) **(A)** Early stage treated with surgical resection. **(B)** Intermediate stage treated with surgical resection. **(C)** Intermediate stage treated with transcatheter arterial chemoembolization (TACE). **(D)** Intermediate stage treated with percutaneous local ablation. **(E)** Intermediate stage treated with systematic therapy. **(F)** Advanced stage treated with supportive care.

We conducted stratified analysis for different BCLC stages between the two periods by the factor of treatments. Because of the censored data in the BCLC very early and the terminal stages, we only compared the remaining data between P1 and P2. For BCLC early stage, the results showed that patients who undertook resection in P2 had longer survival than in P1 (*p* = 0.001 Figure 5A). For patients with BCLC intermediate stage, the patients who underwent more “positive” therapy in P2 lived longer than those in P1 (*p* < 0.001, *p* = 0.001, *p* = 0.008 and *p* = 0.017 for resection, TACE, percutaneous local ablation and systematic therapy, respectively, Figure 5B–5E). Meanwhile, the patients who undertook more “positive” therapy (resection, TACE, percutaneous local ablation and systematic therapy) had much better survival than those who received “conservative” therapy (supportive care and the other therapy) in every period (*p* < 0.001). Considering the BCLC advanced stage, only the patients who received supportive treatment in P2 had longer survival than those in P1 (*p* = 0.002, Figure 5F). Because of the small number of patients, liver transplantation was not analyzed in this stratified analysis.

## DISCUSSION

Our study, for the first time to our knowledge, based on data in a large medical center of northwest of China, evaluated the clinical characteristics, diagnosis, therapy option and survival of patients with HCC over the last ten years. This allowed us to find the changing characteristics regarding HCC in northwestern China. The results of this study showed several changes in clinical features of HCC over the last ten years.

With regard to age, the patients' ages when they were diagnosed grew 1.5 years during the last ten years. This might be attributed to the improvement of general health status in the population and the increasing utilization of antiviral therapy which may delay HCC development in hepatitis virus related patients [14–17]. Unfortunately, we could not evaluate the antiviral effects on the patients with virus infection because of insufficient data. However, the ages of the enrolled patients in our study were younger than those of the patients in studies of USA and Italy [18, 19].

Considering the etiology, of the available records, nearly 80% of the patients are infected with hepatitis virus, and more than 90% of these infections were HBV. HBV infection in HCC remained no obvious change over the 10-year period, but HCV infection increased though both did not show a statistically significant difference. The etiology distribution was different from that in Italy and USA where HCV infection accounts for the major infections [3, 19]. The HBV infection rate in HCC was also much higher than that of several Asian regions such as Japan and Taiwan [19–22]. HBV infection in China was usually acquired from mother-child transmission which would decrease by vaccination [23]. HCV infection accounted for a small proportion of HCC in China where the unsafe blood and blood product transfusion as well as medical operation might mainly attribute to the HCV infection [24]. As the donor screening and the use of disposable medical instruments, the risk of HCV transmission would decline [25]. Meanwhile, the anti-viral treatment would decrease the HCC risk in patients with viral hepatitis [26–28]. It could be anticipated that the hepatitis virus-related HCC may decrease in years to come. In developed countries, alcohol abuse accounts for a high proportion of HCC [19, 29, 30]. We could not evaluate the percentage of tumor caused by alcohol abuse because of the very low proportion of patients with etiological causes other than HBV and HCV and limited information in medical records.

As of the diagnosis of HCC, neither the stages of HCC according to the BCLC staging system nor the grades of Child-Pugh class of the underlying cirrhosis had any significant changes over the 10-year period studied. These findings suggest that the early diagnosis of HCC remains an issue to be resolved although the widespread application of liver imaging including ultrasound, CT and MRI. The reason responsible for the low ratio of early stage patients may be associated to the lacking of an effective surveillance system which is an cost-effective way for patients with HBV or HCV [10]. This phenomenon is consistent with report from Italy where around half of the HCC cases were diagnosed during surveillance with no significant change over a 20-year period [19]. Therefore, aggressive screening programs in high risk populations such as those with HBV and HCV infections and alcohol abuse are required to diagnose HCC at its early stage and to improve patient outcome.

The overall survival rate of HCC patients at 5 years was almost tripled from 10% in P1 to 28% in P2. In addition to the improvement of general health status in the population and the increasing usage of antiviral therapy, the improvement of therapeutics and the changing pattern of treatment options may primarily contribute to the increased survivals in the patients. If the patients in BCLC very early or early stage met the Milan criteria, liver transplantation was a good choice [31]. However, the high cost of transplantation and the shortage of donor organs made the liver transplantation impractical in many cases. Only 26 patients with HCC undertook liver transplantation over last 10 years in our patients. Surgical resection was the recommended therapy for the patients with the BCLC very early and early stage without portal hypertension and abnormal bilirubin [10, 32]. Besides transplantation and resection, radiofrequency ablation and percutaneous alcohol injection were considered choices [33, 34]. Anyway, surgical resection is still the first-line treatment and yields good survival [7]. Our data demonstrated that over the last decade, resection resulted in the best long-term survival improvement for the patients in the BCLC early stage. The five year survival improved from 10% to 45% which was comparable with other studies [32, 35].

Considering patients in the BCLC intermediate stage, our study showed that the “positive” therapies including resection, TACE, percutaneous local ablation and systematic therapy, provided better survival than the conservative therapy (supportive care and the other therapy). These “positive” therapies also showed significant survival improvement during the last ten years. Surprisingly, our study showed that the patients who undertook percutaneous local ablation yielded longer 5 year survival than TACE which was recommended for the BCLC intermediate stage [10]. Our result, however, was similar with another study, indicating that percutaneous local ablation might be another option for BCLC intermediate stage patients [7]. Though a meta-analysis demonstrated that TACE showed beneficial survival [36], a recent review gave no firm evidence to support or refute TACE [37]. Because of the complexity and heterogeneity of BCLC intermediate stage, more studies are needed to assess for a better therapy choice. Sorafenib shows a beneficial survival effect and is recommended as the treatment option for advanced BCLC stage patients [10, 38, 39]. A total of 9 patients who diagnosed as HCC in our hospital received sorafenib since 2007. We, hence, did not assess the effect of sorafenib owing to the very small number of patients. For the supportive therapy, the overall survival improved over the ten years for advanced HCC in our study.

Apart from liver transplantation and surgical resection which are limited by the source of organ and the stage of HCC and the tolerability of patients to operation, respectively, the evolving options of therapeutics in our study were generally related to the efficacy improvement of the treatment over time. The option of TACE had a significant decrease over the 10-year period from P1 to P2, similar to the report from Italy [19]. The survival of patients with TACE was generally lower than that of percutaneous local ablation and systematic therapy and, the survival increase in patients treated with TACE was, although significant, but not as high as that in those treated with percutaneous local ablation and systematic therapy. This may be a reason responsible for the decreased option of TACE. However, the actual reasons associated with the decreased option of TACE in patients at BCLC intermediate stage may be multifactorial. The option of percutaneous local ablation was significantly increased from P1 to P2. The higher survival of percutaneous local ablation in relation to TACE may at least partially explain its increased option. An increasing trend of option for percutaneous local ablation was also shown in the report from Italy [19]. However, the proportion of percutaneous local ablation in our patients was lower than that in the report from Italy [19]. The option of systematic therapy had no significant change but the survival with this therapy had a significant increase. The option of supportive care significantly increased from P1 to P2 with a significant but lower magnitude of increase in survival. The overall survival of supportive therapy had been proved to be inferior to TACE [40]. The option of other therapy had significant reduction from P1 to P2 with no survival improvement. Generally, the treatments with increasing option trend had a higher magnitude of survival increase and *vise versa*. Therefore, the optimal treatment options for every stage of HCC may be a major reason for the increased survival.

Notably, the long-term survival rate in our patients was still lower than that of developed country of comparable years [19]. This might be partially related to the lower ratio of patients diagnosed at very early and early stages in comparison with developed regions [7, 32]. Most HCC patients in China develop the tumor on the underlying liver disease cirrhosis, which may also affect the prognosis of the patients. The patients in our study had lower proportion of Child A and higher proportion of Child B compared with the report from Italy [19]. This may be another reason that the survival in our study is lower than that in other regions [19]. Despite of the tumor status, liver function and general health status, therapy choice determines the prognosis of a patient [5, 10]. Of note, a larger proportion of patients in our study selected conservative therapies, which were associated with lower survival in comparison with the “positive” therapies. Therefore, the therapeutic choice may also partially explicate why the overall survival rates in our patients were lower than those in other study [19].

Our study has limitations. Firstly, it was a retrospective study assessing performance long ago. This might make some assessment inaccurate which would lead to bias. Secondly, some limited data including insufficient antiviral therapy data and limited liver transplant cases made it unable to do some related evaluation. However, our study showed some changing characteristics of HCC, unchanging early diagnosis rate and evolving therapeutic options in patient management during the last ten years in a large medical center of northwest China for the first time. The findings may be helpful for further improving the management of HCC.

In conclusion, to our knowledge, this is the first study involved large data to evaluate the characteristics, diagnosis, survival and treatment options of HCC over a ten-year period in mainland China. In this study, viral infections especially HBV remained the major etiological agents associated with HCC; the rate of patients diagnosed at the early stage of cancer appeared to have no significant increase over a 10-year period; the survival of HCC improved significantly over this 10-year period; the evolving treatment options may be the major factor associated with the survival improvement. The finding that the proportion of patients diagnosed at early stages of HCC was low and did not increase over the last 10 years calls for implementing surveillance system for at risk patients and suggests the potentail to further improve the patient survival by increasing the diagnostic rate of HCC patients at the early stage of disease.

## PATIENTS AND METHODS

### Ethics statements

This study was initiated after receiving approval from the Institutional Review Board of the First Affiliated Hospital, School of Medicine, Xi’an Jiaotong University and carried out according to the Helsinki Declaration.

### Data source

Medical records of patients who were diagnosed HCC between 2002 and 2011 in the First Affiliated Hospital, School of Medicine, Xi’an Jiaotong University were retrospectively reviewed. This hospital has more than 2400 beds for inpatient now. It is the biggest general hospital directly under the administration of the Chinese Ministry of Health in northwest China. The patients who were diagnosed HCC and also treated their disease in this hospital during the study period were included. However, patients who diagnosed their HCC but did not treat the disease in this hospital and those who had comorbidities other than HCC and HCC-related underlying diseases were excluded from the study. For the purpose of comparison and analysis, the data of the eligible patients during the study duration were arbitrarily divided into two periods, 2002–2006 as P1 and 2007–2011 as P2.

### Etiology of HCC

The etiology of liver disease was determined according to the medical records including the medical history provided by patients and laboratory examination.

The etiology was classified as: (1) HBV, if patients were chronically infected with HBV or positive for HBsAg accompanying other HBV seromarkers in laboratory test; (2) HCV, if patients were chronically infected with HCV or had positive anti-HCV antibody in laboratory test; (3) HBV and HCV co-infection, if patients met the first two criteria; (4) Others, if the patients met none of the above criteria.

### Stage of HCC at diagnosis

The HCC stage was classified according to the BCLC staging system which was proved a good HCC prognostic system and recommended by the AASLD guideline [10]. The BCLC staging system classifies the HCC as very early, early, intermediated, advanced, and the terminal stages [13].

### Treatments

The main treatments were classified into 7 categories: (1) liver transplantation; (2) resection; (3) TACE; (4) percutaneous local ablation including percutaneous ablation with ethanol injection or radiofrequency; (5) systematic therapy, treated with at least two of the above treatments except liver transplantation; (6) supportive care and symptomatic treatment; (7) other treatments including systemic chemotherapy, sorafenib, traditional Chinese medicine or conformal radiation therapy.

Liver cirrhosis and tumor metastasis were diagnosed according to medical history or records of liver biopsy, ultrasound, computed tomography (CT) scan and/or magnetic resonance imaging (MRI).

### Endpoint and survival data

Endpoint was HCC associated death. Survival time was defined as the interval time between diagnosed as HCC and the death associated with HCC or the end of 2013. Lost to follow up including died from other disease and survivals at the end of year of 2013 were defined as censored data.

### Statistical analysis

Quantitative data were expressed as mean value ± standard deviation (SD), qualitative data and ordinal data as absolute frequencies. Student's *t* test was used to compare quantitative data between two groups, and chi-square test was used for the qualitative data and ordinal data. Survival curves were analyzed by the Kaplan-Meier method and the log-rank test was used to compare the difference between the subgroups. The survival rates at 1 year, 3 years and 5 years were calculated by life-table method. Statistical analyses were performed using SPSS 21.0 statistical software (SPSS Inc., Chicago, IL, USA). A two-tailed *p* value < 0.05 was considered statistically significant.
